# Deficiency of sphingosine-1-phosphate receptor 3 does not affect the skeletal phenotype of mice lacking sphingosine-1-phosphate lyase

**DOI:** 10.1371/journal.pone.0219734

**Published:** 2019-07-17

**Authors:** Timo Heckt, Laura J. Brylka, Mona Neven, Michael Amling, Thorsten Schinke

**Affiliations:** Department of Osteology and Biomechanics, University Medical Center Hamburg Eppendorf, Hamburg, Germany; Universite de Nantes, FRANCE

## Abstract

Albeit osteoporosis is one of the most prevalent disorders in the aged population, treatment options stimulating the activity of bone-forming osteoblasts are still limited. We and others have previously identified sphingosine-1-phosphate (S1P) as a bone remodeling coupling factor, which is released by bone-resorbing osteoclasts to stimulate bone formation. Moreover, S1pr3, encoding one of the five known S1P receptors (S1P3), was found differentially expressed in osteoblasts, and S1P3 deficiency corrected the moderate high bone mass phenotype of a mouse model (deficient for the calcitonin receptor) with increased S1P release from osteoclasts. In the present study we addressed the question, if S1P3 deficiency would also influence the skeletal phenotype of mice lacking S1P-lyase (encoded by Sgpl1), which display markedly increased S1P levels due to insufficient degradation. Consistent with previous reports, the majority of Sgpl1-deficient mice died before or shortly after weaning, and this lethality was not influenced by additional S1P3 deficiency. At 3 weeks of age, Sgpl1-deficient mice displayed increased trabecular bone mass, which was associated with enhanced osteoclastogenesis and bone resorption, but also with increased bone formation. Most importantly however, none of the skeletal parameters assessed by μCT, histomorphometry and serum analyses were significantly influenced by additional S1P3 deficiency. Taken together, our findings fully support the concept that S1P is a potent osteoanabolic molecule, although S1P3 is not the sole receptor mediating this influence. Since S1P receptors are considered excellent drug targets, it is now required to screen for the impact of other family members on bone formation.

## Introduction

Osteoporosis is one of the most prevalent disorders in the aged population, and skeletal fractures have a high detrimental impact, causing either mortality or reduced quality of life [1]. The disease is caused by a relative increase of bone resorption, mediated by osteoclasts, over bone formation, mediated by osteoblasts [2]. Since the two bone remodeling cells differ with respect to progenitor cells and mode of action, they are also regulated by different sets of molecules. Therefore, the treatment of osteoporosis can either be achieved by blocking differentiation or activity of osteoclasts, i.e. anti-resorptive treatment, and/or by activating bone formation by osteoblasts, i.e. osteoanabolic treatment [3]. Whereas cost-effective anti-resorptives are already available, there is still a need to establish better osteoanabolic treatment options. Moreover, since long-term blockade of osteoclasts can negatively impact bone matrix quality, stimulating bone formation is principally the preferable way to treat osteoporotic patients.

In this regard it is highly relevant that there is evidence for the existence of a molecular crosstalk between osteoblasts and osteoclasts [4–7]. In fact, the two most critical regulators of bone resorption, the cytokine RANKL and its decoy receptor OPG, are both produced by osteoblasts, and a change in the ratio of osteoblast-derived RANKL/OPG specifically affects bone resorption [8]. On the other hand, there is increasing evidence for the existence of osteoclast-derived molecules with a positive influence on bone formation, albeit it remains to be established, which of the molecules proposed to be involved in this coupling process are physiologically relevant. We have previously reported that the hormone calcitonin (CT) mediates a negative influence on bone formation through binding to its receptor (CTR) on osteoclasts [9,10]. At the molecular level this indirect influence is explained by the fact that CT inhibits the osteoclastic expression of Spns2, a transporter facilitating secretion of the osteoanabolic lipid mediator sphingosine-1-phosphate (S1P) [11,12]. We could further show that S1P levels are increased in bone tissue from CT- and CTR-deficient mice, and that the elevated bone formation rate in CTR-deficient mice is normalized by additional deletion of the S1P receptor S1P_3_ [10].

In the course of this study we additionally analyzed the skeletal phenotype of *Sgpl1*^-/-^ mice, which lack the S1P-degrading enzyme S1P-lyase [13,14]. These mice are known to display high extracellular S1P levels causing various organ abnormalities and early postnatal lethality. In all *Sgpl1*-deficient mice reaching the age of 6 weeks (approximately 15%), the trabecular bone mass was remarkably increased, and the same was the case for bone turnover markers in the serum [10]. In the present study we addressed the question, if the high bone turnover phenotype of *Sgpl1*-deficient mice is corrected by additional absence of S1P_3_ (encoded by the *S1pr3* gene). For that purpose we compared the skeletal phenotype of wildtype, *Sgpl1*^*-/-*^ and *Sgpl1*^*-/-*^*/S1pr3*^*-/-*^ mice at 3 weeks of age. Through a combination of μCT analysis, histomorphometry on undecalcified bone sections and serum analyses, we confirmed that *Sgpl1*^*-/-*^ mice display high bone turnover, but also found that the additional absence of S1P_3_ did not affect these abnormalities. This demonstrates that S1P_3_ is not the sole receptor mediating the osteoanabolic influence of S1P *in vivo*.

## Materials and methods

### Animals

*Sgpl1*^*+/-*^ mice were obtained from Jackson Laboratory (#007199), whereas *S1pr3*^*-/-*^ mice were kindly provided by Dr. Jerold Chun, La Jolla, USA [15]. We first generated compound heterozygous mice that were bred to generate *Sgpl1*^*+/+*^, *Sgpl1*^*-/-*^ and *Sgpl1*^*-/-*^/*S1pr3*^*-/-*^littermates. Genotyping for the *Sgpl1* mutation was performed using the primers 5’-CGC TCA GAA GGC TCT GAG TCA TGG-3’, 5’-CCA AGT GTA CCT GCT AAG TTC CAG-3’ and 5’-CAT CAA GGA AAC CCT GGA CTA CTG-3’ to amplify a 300 bp wildtype and/or a 520 bp mutant allele fragment. Genotyping for the *S1pr3* mutation was performed using the primers 5’- CAC AGC AAG CAG ACC TCC AGA -3’, 5’- TGG TGT GCG GCT GTC TAG TCA A -3’ and 5’- ATC GAT ACC GTC GAT CGA CCT -3’ to amplify a 300 bp wildtype and/or a 200 bp mutant allele fragment. All mice were kept with a 12-hour light/dark cycle and had access to tap water and standard rodent chow (1328P, Altromin Spezialfutter GmbH & Co. KG, Germany) *ad libitum*. All animal experiments were approved by the animal facility of the University Medical Center Hamburg-Eppendorf and by the “Behörde für Soziales, Familie, Gesundheit und Verbraucherschutz” (G14/068 and Org529). This also included generation and skeletal analysis of *Sgpl1*-deficient mice. According to the approval the respective mice were scored on a daily basis for apparent abnormalities, such as weight reduction, reduced mobility, abnormal behavior, tumor formation or skeletal fractures. This was performed by trained personnel of the animal facility, together with our own researchers, all of them trained based on FELASA guidelines. According to a respective scoring sheet, animals had to be euthanized to avoid unnecessary suffering. In the case of the born *Sgpl1*-deficient mice, these criteria were not fulfilled at 3 weeks of age, yet a large proportion of them (18 in total) were found dead in the cage before weaning. We therefore decided, also based on the published phenotype [14], to perform our analysis earlier than initially planned and chose 3 weeks of age as a humane endpoint, where all of the analyzed mice (28 in total) were euthanized. Since we did not observe a skeletal phenotype of 3 weeks old *S1pr3*-deficient mice (S1 Fig), consistent with our previous analysis [10], we focused our comparative analysis on wildtype, *Sgpl1*^*-/-*^*/S1pr3*^*+/+*^ and *Sgpl1*^*-/-*^*/S1pr3*^*-/-*^ littermates.

### Histology

Skeletons were fixed in 3.7% PBS-buffered formaldehyde for 24 h and subsequently stored in 80% ethanol. Lungs were embedded into paraffin, and the respective sections were stained with Sirius Red and visualized using polarized light microscopy. For bone histology, the lumbar vertebral bodies L1 to L4 and the right tibia of each mouse were dehydrated in ascending alcohol concentrations and then embedded in methylmetacrylate as described previously [16]. Sections of 4 μm thickness were cut in the sagittal plane on a Microtec rotation microtome (Techno-Med GmbH, Germany) and stained by toluidine blue, or by von Kossa/van Gieson and Goldner staining, as described previously [16]. Histomorphometry was performed according to the ASBMR guidelines [17] using the OsteoMeasure histomorphometry system (Osteometrics Inc., USA).

### μCT analysis

For μCT analysis the right femur of each mouse was fixed and processed as described above. μCT scanning and analysis were performed with a voxel resolution of 10 μm as previously described using a μCT 40 desktop cone-beam microCT (Scanco Medical, Swizerland) according to standard guidelines [18,19]. Trabecular bone was analyzed in the distal metaphysis in a volume situated 2500 μm to 500 μm proximal of the distal growth plate. A threshold value of 250 was implemented.

### Serum analysis

The serum concentrations of the bone turnover markers C-terminal telopeptide of type I collagen (CTX-I, Immunodiagnostic Sytems, #AC-06F1) and C-terminal propeptide of type I procollagen (PICP, Cloud Clone Corp, #SEA570Mu) were determined by ELISA. The same applies for the serum concentrations of the osteoclastogenesis regulators RANKL and OPG (R&D systems, #MTR00; #MOP00).

### Expression analysis

For expression analysis in cortical bone, the femur midshaft was dissected, and the bone marrow was removed by centrifugation. After grinding the bone in liquid nitrogen, RNA was isolated using the RNeasyMini kit (Qiagen), and DNase digestion was performed according to manufacturer’s instructions. Concentration and quality of RNA were measured using a NanoDrop ND-1000 system (NanoDrop Technology). Complimentary DNA synthesis was performed using the Verso cDNA Synthesis Kit (Thermo Fisher). Expression analysis by qRT-PCR was performed using a StepOnePlus system and predesigned TaqMan gene expression assays (Applied Biosystems). *Gapdh* expression was used as an internal control. Relative quantification was performed according to the ΔΔC_T_ method, and results were expressed in the linear form using the formula 2^-ΔΔCT^.

### Statistical analysis

All data presented in the manuscript are presented as means ± standard deviations. Statistical significance was calculated using Mann-Whitney test (Graph Pad Prism), and p-values below 0.05 were considered statistically significant.

## Results

### S1P_3_ deficiency does not prevent lethality of *Sgpl1*-deficient mice

**Fig 1:**
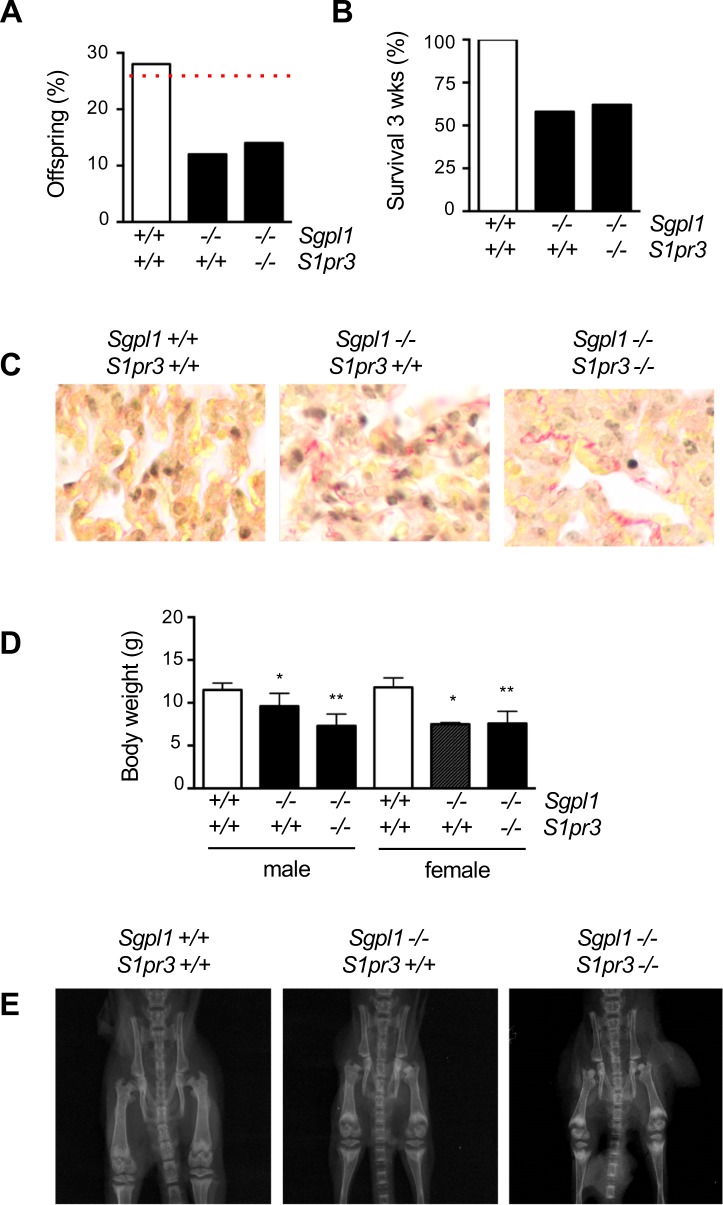
Postnatal lethality of *Sgpl1*-deficient mice is not influenced by additional S1P_3_ deficiency. (A) Percentage of mice genotyped 3–5 days after birth from *Sgpl1*-heterozygous matings. The dotted red line indicates the expected value based on Mendelian inheritance. (B) Percentage of mice of the indicated genotypes that survived until the age of 3 weeks. (C) Representative images of lung sections from 3 weeks old wildtype, *Sgpl1*^*-/-*^*/S1pr3*^*+/+*^ and *Sgpl1*^*-/-*^*/S1pr3*^*-/-*^ mice after sirius red staining for fibrotic tissue. (D) Body weight of 3 weeks old male or female wildtype, *Sgpl1*^*-/-*^*/S1pr3*^*+/+*^ and *Sgpl1*^*-/-*^*/S1pr3*^*-/-*^ mice. (E) Representative contact X-rays of 3 weeks old wildtype, *Sgpl1*^*-/-*^*/S1pr3*^*+/+*^ and *Sgpl1*^*-/-*^*/S1pr3*^*-/-*^ mice. Data represent mean ± SD (n ≥ 5). *p<0.05, **p<0.005 vs. wildtype littermates.

To address the question, if the previously established high bone turnover phenotype of *Sgpl1*-deficient mice is influenced by *S1pr3* deficiency, we generated mice lacking only *Sgpl1* or both genes. We observed that Sgpl1 deficiency, regardless of the *S1pr3* genotype, caused perinatal lethality, i.e. more than 40% of these mice died with the first week after birth (Fig 1A). Since the majority of the surviving mice died either before or shortly after weaning (Fig 1B), we decided to perform our comparative analysis at 3 weeks of age. Here we first analyzed the lung pathology by Sirius Red staining and found a similar extent of fibrotic areas in *Sgpl1*^*-/-*^*/S1pr3*^*+/+*^ and *Sgpl1*^*-/-*^*/S1pr3*^*-/-*^ mice, in agreement with the same lethality rate (Fig 1C). We additionally observed that 3 weeks old *Sgpl1*-deficient mice, both male and female, displayed significantly reduced body weight, and the same was the case in *Sgpl1*^*-/-*^*/S1pr3*^*-/-*^ mice (Fig 1D). As evidenced by contact X-ray, 3 weeks old *Sgpl1*-deficient mice displayed a clear skeletal phenotype, i.e. reduced skeletal growth and increased bone mass, which was apparently unaffected by additional S1P_3_ deficiency (Fig 1E).

### S1P_3_ deficiency does not affect the increased trabecular bone mass phenotype of *Sgpl1*-deficient mice

**Fig 2:**
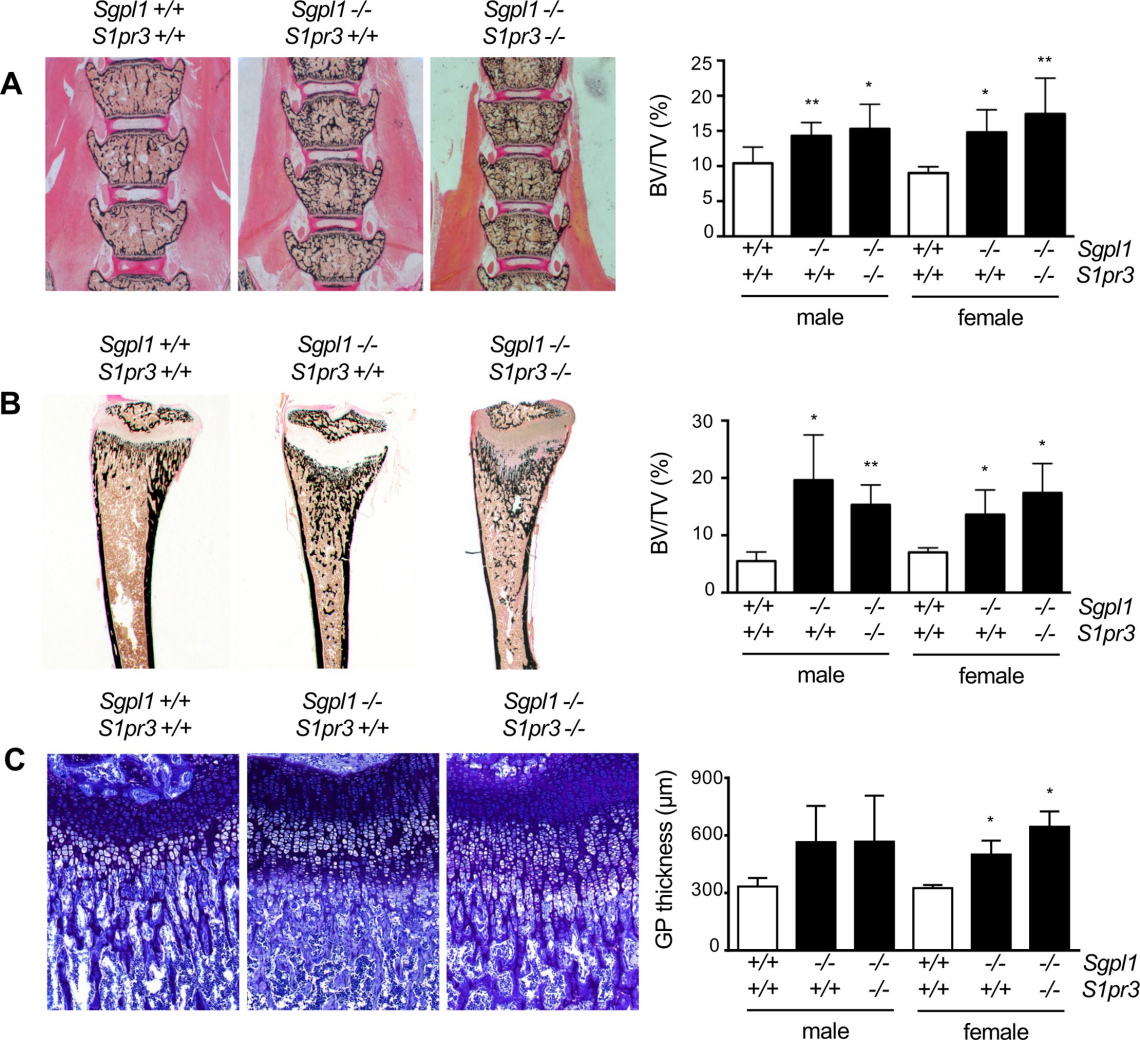
Increased trabecular bone mass of *Sgpl1*-deficient mice is not influenced by additional S1P_3_ deficiency. (A) Representative images of undecalcified spine sections from 3 weeks old male wildtype, *Sgpl1*^*-/-*^*/S1pr3*^*+/+*^ and *Sgpl1*^*-/-*^*/S1pr3*^*-/-*^ mice after von Kossa/van Gieson staining. Mineralized bone appears in black. Quantification of the trabecular bone volume per tissue volume (BV/TV) is given on the right. (B) Representative images of undecalcified tibia sections from the same mice, with quantification of the trabecular bone volume per tissue volume given on the right. (C) Representative images of the same sections after toluidine blue staining. Quantification of the growth plate thickness (GpTh) is given on the right. Data represent mean ± SD (n = 6 males, n ≥ 3 females). *p<0.05, **p<0.005 vs. wildtype littermates.

We next applied undecalcified histology, where we analyzed 3 weeks old male and female groups of mice. In both genders we observed a significant increase of the trabecular bone mass in spine sections of *Sgpl1*-deficient mice, regardless of the *S1pr3* genotype (Fig 2A). The same increase of trabecular bone mass was observed in tibia sections, where an additional widening of the growth plate cartilage was found (Fig 2B). This latter aspect of the phenotype was further confirmed by quantification of the growth plate thickness after toluidine blue staining of the sections. Although there was a high variation in the severity of the phenotype, the growth plate width was significantly increased in female *Sgpl1*-deficient mice, and there was no correction by additional S1P_3_ deficiency (Fig 2C). It is also important to state that the inspection of the subchondral bone areas in these sections revealed that there was no pathological enrichment of cartilage remnants in *Sgpl1*-deficient mice. Since persistence of calcified cartilage is one hallmark of osteopetrosis, a genetic disorder of osteoclast dysfunction [20], the combined histologic analysis confirmed our previous observations from 6 weeks old *Sgpl1*-deficient mice, where the high bone mass phenotype was associated with increased bone formation and resorption [10].

**Fig 3:**
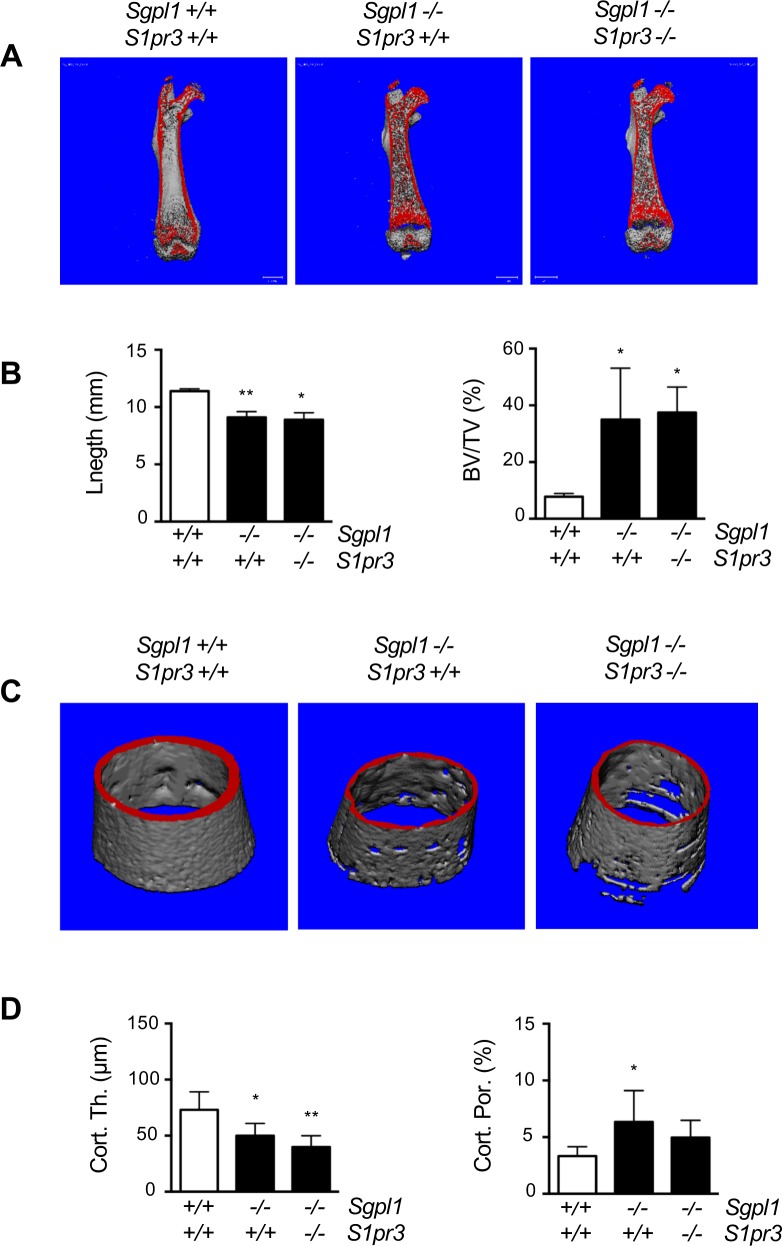
Changes in trabecular and cortical bone of *Sgpl1*-deficient mice are not influenced by additional S1P_3_ deficiency. (A) Representative μCT images of femora from 3 weeks old male wildtype, *Sgpl1*^*-/-*^*/S1pr3*^*+/+*^ and *Sgpl1*^*-/-*^*/S1pr3*^*-/-*^ mice. Mineralized bone appears in red. (B) Quantification of the femoral length and the trabecular bone volume per tissue volume (BV/TV) in the three groups of mice. (C) Representative μCT images of the femoral midshaft from the same mice. (D) Quantification of cortical thickness (Cort. Th.) and porosity (Cort. Por.) in the three groups of mice. Data represent mean ± SD (n = 5). *p<0.05, **p<0.005 vs. wildtype littermates.

We additionally applied μCT scanning of femoral bones, where we focused on male mice (Fig 3A). Here we observed a significant length reduction in both, *Sgpl1*^*-/-*^*/S1pr3*^*+/+*^ and *Sgpl1*^*-/-*^*/S1pr3*^*-/-*^ animals. Moreover, the *Sgpl1*-deficient mice displayed an increased trabecular bone volume which was unaffected by additional S1P_3_ deficiency (Fig 3B). We also identified changes in the cortical bone compartment. More specifically, cortical bone mass was decreased in *Sgpl1*-deficient mice, which was associated with higher cortical porosity, indicative of increased bone resorption (Fi. 3C). Although there was no significant difference between wildtype and *Sgpl1*^*-/-*^*/S1pr3*^*-/-*^ mice in the latter parameter, the cortical porosity in *Sgpl1*-deficient femora was not significantly decreased by additional S1P_3_ deficiency (Fig 3D).

### High bone turnover in Sgpl1-deficient mice is not corrected by S1P3 deficiency

**Fig 4:**
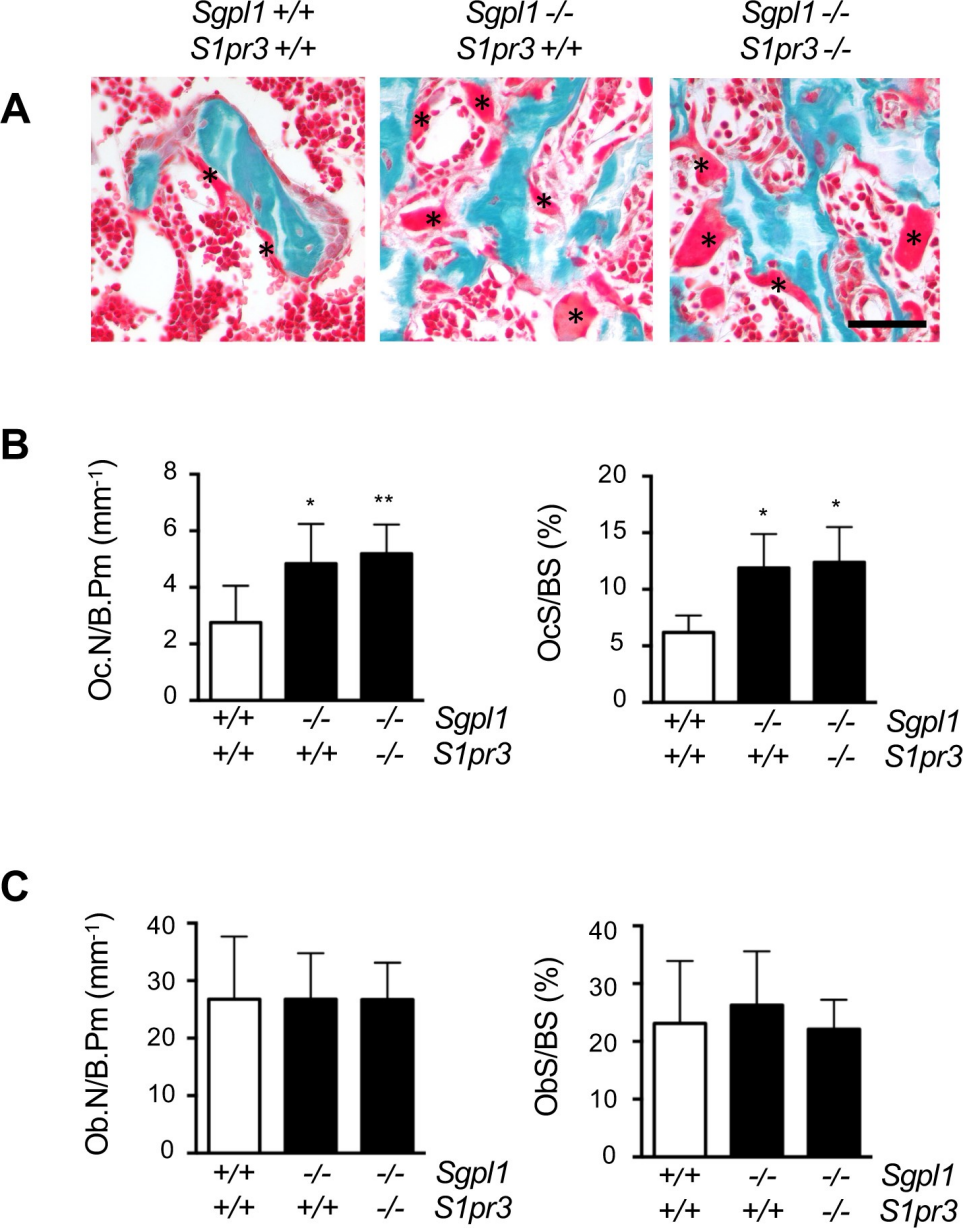
Increased osteoclastogenesis of *Sgpl1*-deficient mice is not influenced by additional S1P_3_ deficiency. (A) Representative images of undecalcified spine sections from 3 weeks old male wildtype, *Sgpl1*^*-/-*^*/S1pr3*^*+/+*^ and *Sgpl1*^*-/-*^*/S1pr3*^*-/-*^ mice after Goldner staining. Mineralized bone appears in green, osteoclasts are highlighted by asterisks. (B) Histomorphometric quantification of osteoclast number (Oc.N./B.Pm, osteoclast number per bone perimeter) and surface (OcS/BS, osteoclast surface per bone surface) in the same mice. (C) Histomorphometric quantification of osteoblast number (Ob.N./B.Pm, osteoclast number per bone perimeter) and surface (ObS/BS, osteoclast surface per bone surface) in the same mice. Data represent mean ± SD (n = 5). *p<0.05, **p<0.005 vs. wildtype littermates.

We finally quantified number and activities of bone cell types by cellular histomorphometry and serum analyses. As evidenced by Goldner staining of spine sections, there was a remarkable increase of osteoclastogenesis in Sgpl1-deficient mice (Fig 4A). This was confirmed by histomorphometry, where we found significantly increased osteoclast parameters (Oc.N/B.Pm, osteoclast number per bone perimeter; OcS/BS, osteoclast surface per bone surface) in both, *Sgpl1*^*-/-*^*/S1pr3*^*+/+*^ and *Sgpl1*^*-/-*^*/S1pr3*^*-/-*^ mice (Fig 4B). In contrast, we did not observe significant differences between the three groups of mice for osteoblast parameters (Ob.N/B.Pm, osteoblast number per bone perimeter; ObS/BS, osteoblast surface per bone surface) (Fig 4C), suggesting that osteoblast differentiation is not affected by S1P.

**Fig 5:**
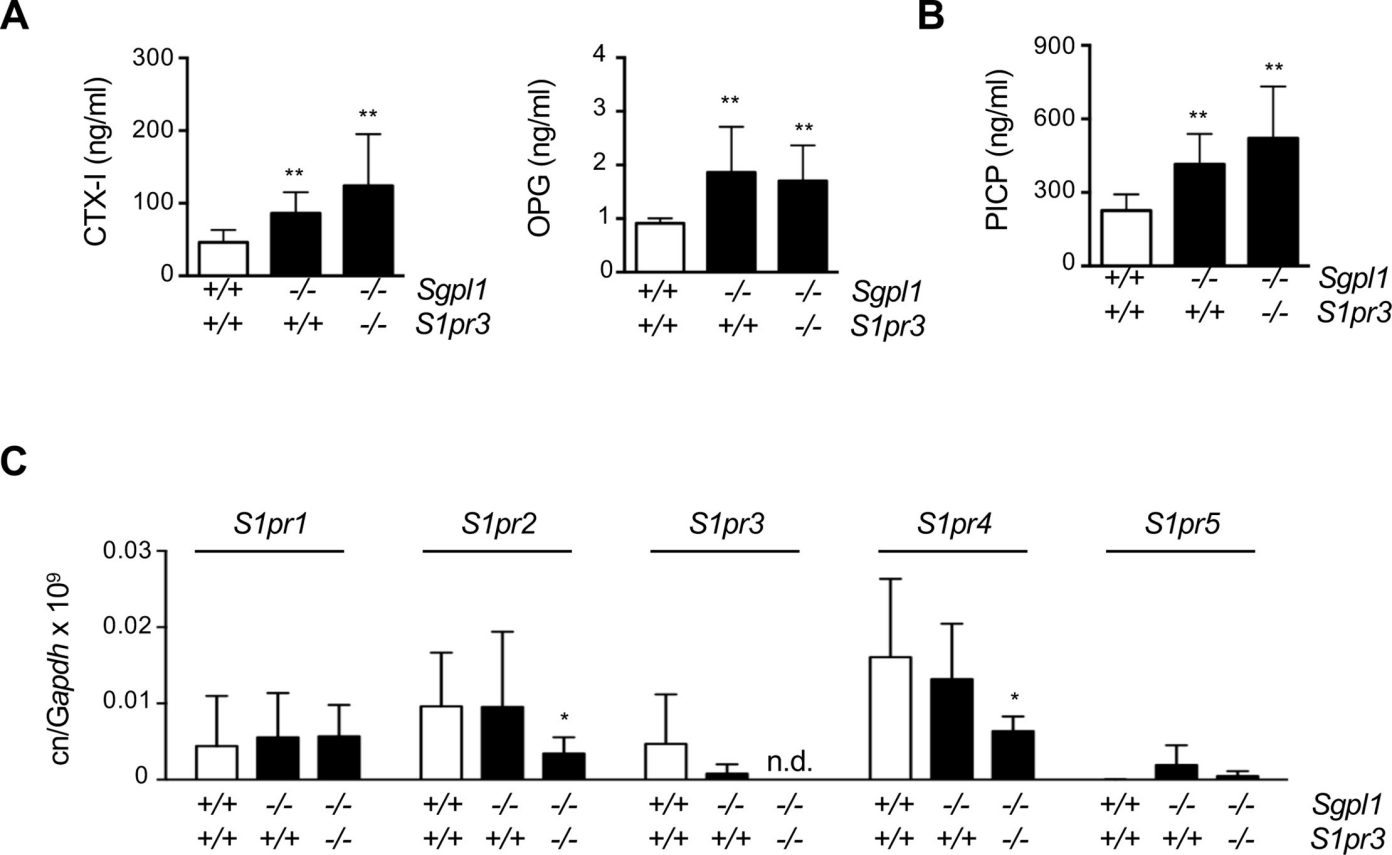
The high bone turnover phenotype of *Sgpl1*-deficient mice is not influenced by additional S1P_3_ deficiency. (A) CTX-I and OPG concentrations in sera from 3 weeks old wildtype, *Sgpl1*^*-/-*^*/S1pr3*^*+/+*^ and *Sgpl1*^*-/-*^*/S1pr3*^*-/-*^ mice. (B) PICP concentrations in sera from the same mice. (C) qRT-PCR monitoring of the expression of the indicated genes in bones of 3 weeks old male wildtype, *Sgpl1*^*-/-*^*/S1pr3*^*+/+*^ and *Sgpl1*^*-/-*^*/S1pr3*^*-/-*^ mice. Data represent mean ± SD (n ≥ 6). *p<0.05, **p<0.005 vs. wildtype littermates.

Since histomorphometric quantification of bone cell functions is difficult for growing mice, we took advantage of established biomarker assays to determine the bone status in the different groups of animals. Serum levels of the bone resorption biomarker CTX-I were elevated in *Sgpl1*-deficient mice, regardless of the *S1pr3* genotype, thereby confirming that the osteoclast population in the *Sgpl1*-deficient mouse model is not functionally impaired (Fig 5A). Of note, serum concentrations of osteoprotegerin (OPG), a physiological inhibitor of osteoclastogenesis [8], whose expression was recently found to be induced by S1P [21], were also elevated in both, *Sgpl1*^*-/-*^*/S1pr3*^*+/+*^ and *Sgpl1*^*-/-*^*/S1pr3*^*-/-*^ mice. Finally, serum concentrations of the bone formation biomarker PICP were significantly increased in *Sgpl1*-deficient mice, thus confirming that S1P promotes osteoblast activity [10]. Most importantly, PICP levels were not significantly different between *Sgpl1*^*-/-*^*/S1pr3*^*+/+*^ and *Sgpl1*^*-/-*^*/S1pr3*^*-/-*^ mice (Fig 5B).

To analyze for a potential compensatory induction of other S1P receptors in *Sgpl1*^*-/-*^*/S1pr3*^*-/-*^ mice, we additionally determined the expression of the five known *S1pr* genes in bones from wildtype, *Sgpl1*^*-/-*^*/S1pr3*^*+/+*^ and *Sgpl1*^*-/-*^*/S1pr3*^*-/-*^ mice. We did not observe a higher level of expression for any of the *S1pr* genes in *Sgpl1*^*-/-*^*/S1pr3*^*-/-*^ mice, when compared to *Sgpl1*-deficient mice (Fig 5C). There was however a significant reduction in the expression of *S1pr2* and *S1pr4* in *Sgpl1*^*-/-*^*/S1pr3*^*-/-*^ bones, when compared to wildtype controls. Apparently, this difference was rather moderate, and since the expression levels were generally low (in some samples not detectable), there was high overall variation within the data sets. Nevertheless, since there was no induction of a specific *S1pr* gene in the absence of S1P_3_, we can only speculate about the relevance of other S1P receptors in the context of osteoanabolic influences.

## Discussion

The lipid mediator S1P has various functions in development, immunity and different homeostatic processes [22–24]. Its synthesis depends on the activity of sphingosine kinases (Sphk1 or Sphk2), whereas its secretion is mediated by the S1P transporter Spns2. The cellular effects of extracellular S1P are transmitted through five G-protein coupled receptors (S1P_1-5_), which are generally considered excellent drug targets [25–27]. Finally, whereas S1P signaling can be inactivated by different lipid phosphatases, irreversible S1P degradation is facilitated by the activity of S1P lyase [28]. The major relevance of S1P lyase for various physiological processes is fully supported by the phenotype of *Sgpl1*-deficient mice, which not only display lymphopenia, but also histological abnormalities in different organs and a markedly reduced life span [14]. Of note, given the baseline characterization of *Sgpl1*^*-/-*^*/S1pr3*^*+/+*^ and *Sgpl1*^*-/-*^*/S1pr3*^*-/-*^ mice described here, we can certainly rule out that S1P_3_ is the major driver of S1P-mediated toxicity.

An influence of S1P on bone cells was first uncovered by the finding that *Sphk1* expression increases during osteoclastogenesis, whereas extracellular S1P promotes osteoblast differentiation *in vitro* [29,30]. The physiological relevance of S1P as a coupling factor in bone remodeling was further demonstrated in two mouse models displaying increased bone formation. Whereas osteoclast-specific deletion of cathepsin K, a matrix-degrading enzyme required for bone resorption, triggered an increased expression of *Sphk1* in osteoclasts [31], our own analysis of mice lacking the CTR demonstrated that the action of CT as a negative regulator of bone formation is molecularly explained by inhibition of *Spns2* expression, thereby reducing S1P release from osteoclasts [10]. Of note, there is also *in vitro* evidence for an influence of S1P on chondrocytes [32]. More specifically, the finding that S1P promotes the proliferation of primary rat chondrocytes in an Erk-dependent manner [33], may explain the expansion of the growth plate in *Sgpl1*-deficient mice. Since this aspect of the phenotype was however not influenced by S1P_3_ deficiency we did not further focus on the skeletal growth phenotype of *Sgpl1*-deficient mice.

In fact, the primary question of our study was if S1P_3_ deficiency is the major mediator of the osteoanabolic influence of S1P. Our specific focus on S1P_3_ is based on our previous study, where we identified S1P as a coupling factor, whose secretion by osteoclasts is regulated by CT [10]. In this study we screened for the expression of all S1P receptors in cultured osteoblasts and identified two candidates (S1P_1_ and S1P_3_) with differential expression during osteoblastogenesis. We subsequently analyzed the skeletal phenotypes of mice lacking S1P_1_ specifically in osteoblasts (*S1pr1*^*fl/fl;Runx2-Cre*^) or S1P_3_ ubiquitously (*S1pr3*^*-/-*^). Whereas *S1pr1*^*fl/fl;Runx2-Cre*^ mice did not display a bone mass phenotype, a reduction of bone formation was observed in 8 months old *S1pr3*-deficient mice. We also found that S1P_3_ deficiency corrected the high bone mass phenotype of CTR-deficient mice and abolished the osteoanabolic effect of the non-selective S1P receptor agonist FTY720 [10]. Therefore, since S1P receptors are generally considered excellent drug targets, it was important to analyze the impact of S1P_3_ deficiency on the skeletal phenotype of *Sgpl1*-deficient mice. Here the most important parameter was related to bone formation, since cost-effective osteoananbolic treatment options still need to be established.

Through a combination of μCT, undecalcified histology, bone-specific histomorphometry and serum analyses, we could clearly demonstrate that 3 weeks old *Sgpl1*-deficient mice display a high bone mass phenotype due to increased osteoblast activity. In comparison to our previous study [10] it is important state that we formerly observed a two-fold increase of the trabecular bone mass in spine sections of 6 weeks old *Sgpl1*-deficient mice, yet there was no significant difference between 3 weeks old male wildtype and *Sgpl1*-littermates (n = 3). Although we can only speculate about the reason for the earlier onset phenotype in the present study, which may relate to a change in the laboratory diet or a modified genetic background, it is important to state that we generally use littermates from heterozygous matings for comparative analyses. It is also relevant that we did not only increase the number of 3 weeks old mice in the present study, but also extended our analysis to tibia sections, μCT imaging and serum biomarkers. Therefore, our major conclusion, i.e. that the high bone formation phenotype of *Sgpl1*-deficient mice is not corrected by S1P_3_ deficiency, is validated by the present data. Similarly, the increased osteoclastogenesis of *Sgpl1*-deficient mice, which occurred despite increased production of the anti-osteoclastogenic molecule OPG, was not different in *Sgpl1*^*-/-*^*/S1pr3*^*-/-*^ mice. Taken together, these findings undoubtedly demonstrate that S1P_3_ is not the sole receptor that responds to markedly increased S1P levels, caused by S1P lyase deficiency, to translate them into increased osteoblast activity. This raises the question, if one of the other S1P receptors is involved in mediating the striking osteoanabolic influence of extracellular S1P, or if the skeletal phenotype of *Sgpl1*-deficient mice is only correctable by combined S1P receptor deficiencies. Unfortunately, this question is difficult to address in mice with ubiquitous deficiency of S1P lyase, given their early postnatal lethality. Moreover, as 3 weeks old mice are characterized by rapid formation and modeling of the bone matrix, whereas remodeling is much more relevant for bone repair in adult mice, there is another major limitation of the *Sgpl1*-deficient mouse model.

Therefore, since the potential impact of S1P on bone remodeling cannot be studied in 3 weeks old mice, it was important that another model was established during the course of our present study. Here it was reported that deletion of S1P lyase in adult mice, achieved by an inducible Cre-loxP system, causes increased trabecular and cortical bone mass [21]. These data are consistent with our study of growing *Sgpl1*-deficient mice in terms of bone formation and serum OPG levels. However, in contrast to what we observed in the present manuscript (i.e. increased osteoclastogenesis and bone resorption in *Sgpl1*-deficient mice), the inducible inactivation of S1P lyase in adult mice resulted in decreased bone resorption [21]. Regardless of this age-dependent inconsistency, the combined data on the action of S1P on bone cells provide strong evidence for the probability to treat osteoporosis by selective S1PR agonists. In this regard, it is important so state that the molecular influence of S1P on cultured osteoblasts was also found to be abolished by antagonism or deficiency of S1P_2_ [21]. Moreover, since *S1pr2*-deficient mice, similar to *S1pr3*-deficient mice [10] were found to display osteopenia, it might be informative to combine the S1P_2_-deficiency with mouse models of increased S1P levels (including *Sgpl1*-deficient mice) in future experiments.

## Supporting information

S1 Fig *S1pr3-*deficient mice do not display a skeletal phenotype at 3 weeks of age. (A) Representative contact X-rays of 3 weeks old wildtype and *S1pr3*^*-/-*^ mice. (B) Representative images of undecalcified spine sections from 3 weeks old wildtype and *S1pr3*^*-/-*^ mice after von Kossa/van Gieson staining. (C) Quantification of the trabecular bone volume per tissue volume (BV/TV) in spine sections of 3 weeks old male or female wildtype, and *S1pr3*^*-/-*^ mice (n ≥ 5). (D) CTX-I concentrations in sera from the same mice.
(TIF)

## References

[1] Kanis JA, Odén A, McCloskey E V., Johansson H, Wahl DA, Cooper C, et al A systematic review of hip fracture incidence and probability of fracture worldwide. Osteoporos Int. 2012;23: 2239–2256. 10.1007/s00198-012-1964-3 22419370 PMC3421108

[2] Zaidi M. Skeletal remodeling in health and disease. Nat Med. 2007;13: 791–801. 10.1038/nm1593 17618270

[3] Rachner TD, Hadji P, Hofbauer LC. Novel therapies in benign and malignant bone diseases. Pharmacol Ther. 2012;134: 338–44. 10.1016/j.pharmthera.2012.02.005 22401778

[4] Del Fattore A, Teti A, Rucci N. Bone cells and the mechanisms of bone remodelling. Front Biosci (Elite Ed). 2012;4: 2302–21. 22202038 10.2741/e543

[5] Henriksen K, Karsdal MA, John Martin T. Osteoclast-Derived Coupling Factors in Bone Remodeling. Calcif Tissue Int. 2014;94: 88–97. 10.1007/s00223-013-9741-7 23700149

[6] Sims NA, Martin TJ. Coupling Signals between the Osteoclast and Osteoblast: How are Messages Transmitted between These Temporary Visitors to the Bone Surface? Front Endocrinol (Lausanne). 2015;6: 41. 25852649 10.3389/fendo.2015.00041 PMC4371744

[7] Ikeda K, Takeshita S. Factors and Mechanisms Involved in the Coupling from Bone Resorption to Formation: How Osteoclasts Talk to Osteoblasts. J Bone Metab. 2014;21: 163 10.11005/jbm.2014.21.3.163 25247154 PMC4170079

[8] Lacey DL, Boyle WJ, Simonet WS, Kostenuik PJ, Dougall WC, Sullivan JK, et al Bench to bedside: elucidation of the OPG–RANK–RANKL pathway and the development of denosumab. Nat Rev Drug Discov. 2012;11: 401–419. 10.1038/nrd3705 22543469

[9] Hoff AO, Catala-Lehnen P, Thomas PM, Priemel M, Rueger JM, Nasonkin I, et al Increased bone mass is an unexpected phenotype associated with deletion of the calcitonin gene. J Clin Invest. 2002;110: 1849–1857. 10.1172/JCI200214218 12488435 PMC151647

[10] Keller J, Catala-Lehnen P, Huebner AK, Jeschke A, Heckt T, Lueth A, et al Calcitonin controls bone formation by inhibiting the release of sphingosine 1-phosphate from osteoclasts. Nat Commun. 2014;5: 5215 10.1038/ncomms6215 25333900 PMC4205484

[11] Kawahara A, Nishi T, Hisano Y, Fukui H, Yamaguchi A, Mochizuki N. The Sphingolipid Transporter Spns2 Functions in Migration of Zebrafish Myocardial Precursors. Science (80-). 2009;323: 524–527. 19074308 10.1126/science.1167449

[12] Hisano Y, Kobayashi N, Yamaguchi A, Nishi T. Mouse SPNS2 Functions as a Sphingosine-1-Phosphate Transporter in Vascular Endothelial Cells. Holowka D, editor. PLoS One. 2012;7: e38941 10.1371/journal.pone.0038941 22723910 PMC3379171

[13] Liu X, Zhang Q-H, Yi G-H. Regulation of metabolism and transport of sphingosine-1-phosphate in mammalian cells. Mol Cell Biochem. 2012;363: 21–33. 10.1007/s11010-011-1154-1 22113622

[14] Vogel P, Donoviel MS, Read R, Hansen GM, Hazlewood J, Anderson SJ, et al Incomplete inhibition of sphingosine 1-phosphate lyase modulates imune system function yet prevents early lethality and non-lymphoid lesions. Alberola-Ila J, editor. PLoS One. 2009;4: e4112 10.1371/journal.pone.0004112 19119317 PMC2606024

[15] Ishii I, Friedman B, Ye X, Kawamura S, McGiffert C, Contos JJA, et al Selective Loss of Sphingosine 1-Phosphate Signaling with No Obvious Phenotypic Abnormality in Mice Lacking Its G Protein-coupled Receptor, LP B3/EDG-3. J Biol Chem. 2001;276: 33697–33704. 10.1074/jbc.M104441200 11443127

[16] Albers J, Keller J, Baranowsky A, Beil FT, Catala-Lehnen P, Schulze J, et al Canonical Wnt signaling inhibits osteoclastogenesis independent of osteoprotegerin. J Cell Biol. 2013;200: 537–549. 10.1083/jcb.201207142 23401003 PMC3575535

[17] Parfitt AM, Drezner MK, Glorieux FH, Kanis JA, Malluche H, Meunier PJ, et al Bone histomorphometry: Standardization of nomenclature, symbols, and units: Report of the asbmr histomorphometry nomenclature committee. J Bone Miner Res. 2009;2: 595–610. 10.1002/jbmr.5650020617 3455637

[18] Yorgan TA, Peters S, Jeschke A, Benisch P, Jakob F, Amling M, et al The Anti-Osteoanabolic Function of Sclerostin Is Blunted in Mice Carrying a High Bone Mass Mutation of Lrp5. J Bone Miner Res. 2015;30: 1175–1183. 10.1002/jbmr.2461 25640331

[19] Bouxsein ML, Boyd SK, Christiansen BA, Guldberg RE, Jepsen KJ, Müller R. Guidelines for assessment of bone microstructure in rodents using micro-computed tomography. J Bone Miner Res. 2010;25: 1468–1486. 10.1002/jbmr.141 20533309

[20] Sobacchi C, Schulz A, Coxon FP, Villa A, Helfrich MH. Osteopetrosis: genetics, treatment and new insights into osteoclast function. Nat Rev Endocrinol. 2013;9: 522–536. 10.1038/nrendo.2013.137 23877423

[21] Weske S, Vaidya M, Reese A, von Wnuck Lipinski K, Keul P, Bayer JK, et al Targeting sphingosine-1-phosphate lyase as an anabolic therapy for bone loss. Nat Med. 2018;24: 667–678. 10.1038/s41591-018-0005-y 29662200

[22] Mendelson K, Evans T, Hla T. Sphingosine 1-phosphate signalling. Development. 2014;141: 5–9. 10.1242/dev.094805 24346695 PMC3865745

[23] Don-Doncow N, Zhang Y, Matuskova H, Meissner A. The emerging alliance of sphingosine-1-phosphate signalling and immune cells: from basic mechanisms to implications in hypertension. Br J Pharmacol. 2018; 10.1111/bph.14381 PMC6534782 29856066

[24] Spiegel S, Milstien S. Sphingosine-1-phosphate: an enigmatic signalling lipid. Nat Rev Mol Cell Biol. 2003;4: 397–407. 10.1038/nrm1103 12728273

[25] Chun J, Hla T, Lynch KR, Spiegel S, Moolenaar WH. International Union of Basic and Clinical Pharmacology. LXXVIII. Lysophospholipid Receptor Nomenclature. Pharmacol Rev. 2010;62: 579–587. 10.1124/pr.110.003111 21079037 PMC2993255

[26] Mutoh T, Rivera R, Chun J. Insights into the pharmacological relevance of lysophospholipid receptors. Br J Pharmacol. Wiley-Blackwell; 2012;165: 829–44. 10.1111/j.1476-5381.2011.01622.x 21838759 PMC3312481

[27] Overington JP, Al-Lazikani B, Hopkins AL. How many drug targets are there? Nat Rev Drug Discov. 2006;5: 993–996. 10.1038/nrd2199 17139284

[28] Van Veldhoven PP. Sphingosine-1-phosphate lyase. Methods Enzymol. 2000;311: 244–54. 10563331 10.1016/s0076-6879(00)11087-0

[29] Pederson L, Ruan M, Westendorf JJ, Khosla S, Oursler MJ. Regulation of bone formation by osteoclasts involves Wnt/BMP signaling and the chemokine sphingosine-1-phosphate. Proc Natl Acad Sci. 2008;105: 20764–20769. 10.1073/pnas.0805133106 19075223 PMC2603259

[30] Ryu J, Kim HJ, Chang E-J, Huang H, Banno Y, Kim H-H. Sphingosine 1-phosphate as a regulator of osteoclast differentiation and osteoclast–osteoblast coupling. EMBO J. 2006;25: 5840–5851. 10.1038/sj.emboj.7601430 17124500 PMC1698879

[31] Lotinun S, Kiviranta R, Matsubara T, Alzate JA, Neff L, Lüth A, et al Osteoclast-specific cathepsin K deletion stimulates S1P-dependent bone formation. J Clin Invest. 2013;123: 666–81. 10.1172/JCI64840 23321671 PMC3561821

[32] Khavandgar Z, Murshed M. Sphingolipid metabolism and its role in the skeletal tissues. Cell Mol Life Sci. 2015;72: 959–969. 10.1007/s00018-014-1778-x 25424644 PMC11114007

[33] Kim M-K, Lee HY, Kwak J-Y, Park J-I, Yun J, Bae Y-S. Sphingosine-1-phosphate stimulates rat primary chondrocyte proliferation. Biochem Biophys Res Commun. 2006;345: 67–73. 10.1016/j.bbrc.2006.04.042 16674917

