# Supplementary figures and images for: Deficiency of sphingosine-1-phosphate receptor 3 does not affect the skeletal phenotype of mice lacking sphingosine-1-phosphate lyase
Source: PLoS One. 2019 Jul 17;14(7):e0219734. doi: 10.1371/journal.pone.0219734 (PMC6636735; doi:10.1371/journal.pone.0219734)

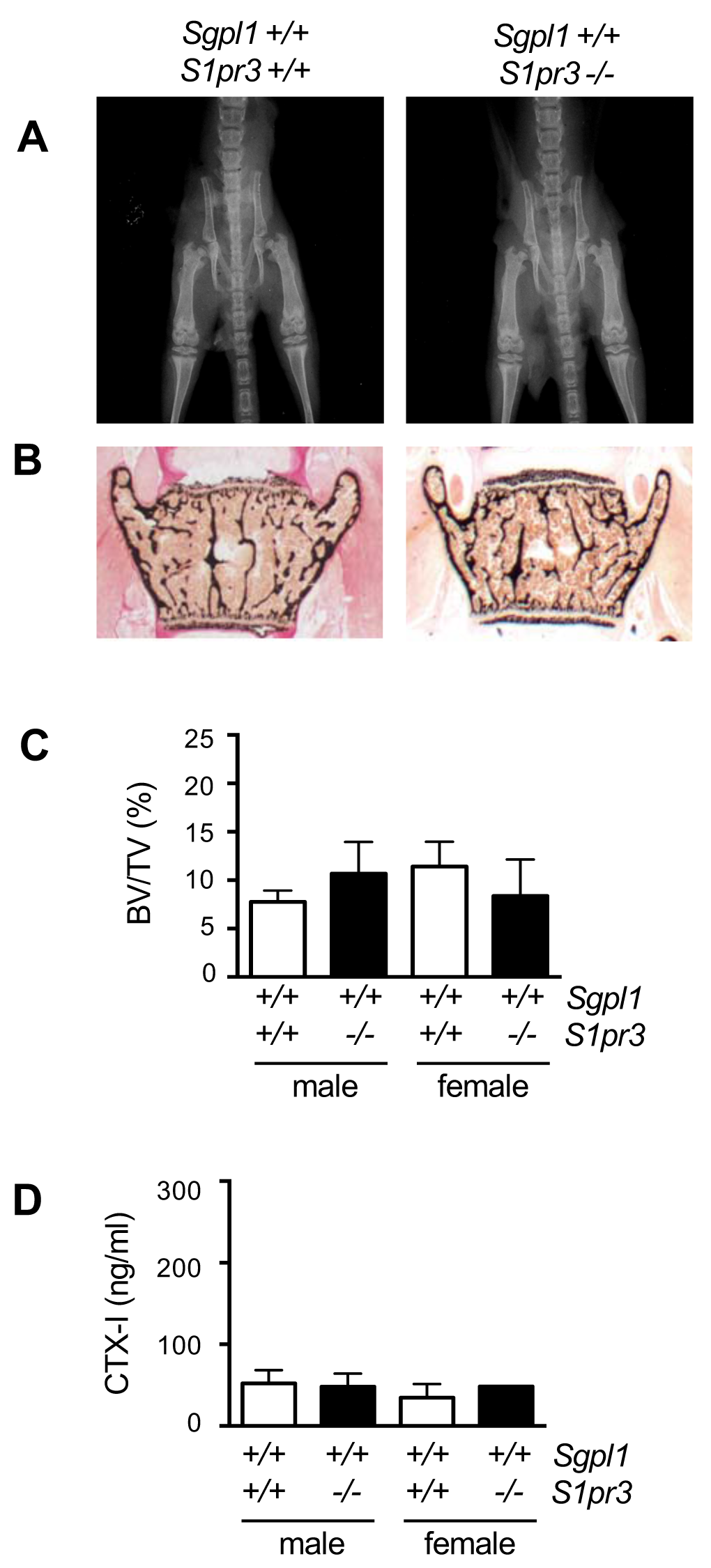

Supplement: S1 Fig — (A) Representative contact X-rays of 3 weeks old wildtype and S1pr3-/- mice. (B) Representative images of undecalcified spine sections from 3 weeks old wildtype and S1pr3-/- mice after von Kossa/van Gieson staining. (C) Quantification of the trabecular bone volume per tissue volume (BV/TV) in spine sections of 3 weeks old male or female wildtype, and S1pr3-/- mice (n ≥ 5). (D) CTX-I concentrations in sera from the same mice. (TIF) [file pone.0219734.s001.tif]
